# The validity of utilizing karate in youth safety education

**DOI:** 10.3389/fspor.2024.1400920

**Published:** 2024-05-30

**Authors:** Paweł Adam Piepiora, Robert Gwardyński

**Affiliations:** Faculty of Physical Education and Sports, Wroclaw University of Health and Sport Sciences, Wrocław, Poland

**Keywords:** karate culture, combat sport, martial art, self-defence system, safety

## Abstract

This article aims to present a new argument on the validity of utilizing karate in youth safety education. First, the important role of safety education and the need to seek new means of influencing young people is presented and the significant role of sport in this area. The field of Far Eastern combat sports was underlined here as being particularly important because they contain internal codes of moral conduct. Therefore, the new argument is to take the perspective of karate as a combat sport, martial art, and self defence system in one. Karate as a combat sport refers to fighting skills in the following directions: traditional, sport and Olympic; and in the following systems: semi contact, knockdown, full contact, mix fighting. Karate as a martial art has a health-promoting character and emphasises the psycho-physical development of practitioners and the recognition of ethical codes. And karate as a self defence system is concerned with proficiency in out-of-sport confrontations, but also in risk assessment. Karate has been found to be an effective means in educating young people to safety, but in order for it to be effective, it needs to be adopted in its entirety. Because the combat sport perspective activates youth in physical culture, the martial art perspective educates according to norms and rules; and the self defence system perspective teaches how to act in difficult situations.

## Introduction

It is human nature to provide security for oneself, one's loved ones and the community within which the individual functions. Therefore, within the realm of education, education for security can be distinguished, which serves to prepare people to take appropriate individual or collective actions in the face of threats to the state and its citizens (1). In practice, education for security is mainly implemented by education system, state and private institutions, as well as associations and non-governmental organisations (2). The priority here is to raise public awareness of the issues of understanding security threats and to develop competences to respond to them in a targeted and rational manner (3).

From point of view it can be seen that the priority in safety education for young people is to shape their cognitive and performance skills. But rationally planned and conducted classes should also take into account the formation of the emotional-motivational sphere by developing pro-social attitudes based on commonly accepted cultural values (4). With this in mind, it makes sense to seek various means of influencing people so that they not only know how to act and are able to do so with a proficiency that is appropriate to the emergency, but also want to do so both individually and with others in the spirit of compliance and discipline in a situation of grave danger. It is therefore right that education for youth safety should draw on physical culture taking the form of physical activity in the broadest sense with a particular focus on sport (5).

Countering threats requires a certain fitness of the body as a tool for all action. Some sporting disciplines prepare more than others for security tasks. These are disciplines that have a particular utilitarian dimension, deriving almost directly from that sphere of human activity which consisted in satisfying the need for safety and preventing injury or loss of life caused by unlawful human behaviour, animal attack, natural disasters or technical failures. Such disciplines can include those in which competition does not involve direct contact (e.g., long-distance running, swimming, shooting, archery), as well as those in which winning a fight involves physical contact (e.g., combat sports, rugby, American football). It is important to emphasize here the special role of combat sports originating from the Far East, as they incorporate internal codes of moral conduct (judo, ju-jitsu, karate, taekwondo, etc.). Admittedly, the main goal of combat sports is to perform as well as possible in a sporting confrontation, but in the case of the Far Eastern varieties it is also important to maintain physical and mental health (because every Far Eastern combat sport is martial art) and non-sporting confrontation skills (the utilitarianism of combat sport as a self-defence system) (6). This is undoubtedly an asset in educating for the safety of young generations and therefore this area of physical activity should be considered appropriate.

Fighting arts researchers (7, 8) are not unanimous as to the validity of a single overarching combat method for safety education. Versatility is recommended, but this is not consistent with the codes of the various Far Eastern combat sports. The solution to this problem is a modern approach to karate (9). There are many style variations in karate, and the masters of the respective schools shape their training goals differently. For some, the most important thing is sporting performance in seasonal competitions−this is the perspective of karate as a combat sport. For others, the most important thing is to develop and maintain physical and mental fitness−this is the martial art perspective. For yet others, the most important thing is to develop skills in non-sport combat−and this, in turn, is the self-defence system perspective (10). Therefore, the aim of this article is to present the validity of utilizing karate for safety education.

## Benefits of karate as a combat sport

It should be noted that karate competition takes place in three strands: traditional, sport and Olympic (11). Competitions in traditional karate are held within the style rules of shobu ippon. These are style-defined bouts that can be won by scoring an ippon or two waza ari. An ippon is awarded for performing such a technique on an opponent that, in an out-of-sport confrontation, would knock him down. A waza ari, on the other hand, is awarded for performing such a technique on an opponent that, in an out-of-sport confrontation, would damage the opponent (12). In the sport karate strand, on the other hand, fights are timed by a particular federation. Kicks to the opponent's head and uppercuts with an opponent's punch are scored higher than kicks to the opponent's torso and punches to the opponent. The competitor who has more points at the end of the regulation time of the bout wins (13). The Olympic karate strand, on the other hand, is defined by the rules of the World Karate Federation and is approved by the International Olympic Committee. In combat, techniques are scored according to the difficulty of execution: ippon is awarded for a kick to the opponent's head or for a scored technique on a lying opponent; waza ari is awarded for a kick to the opponent's torso; yuko is awarded for striking the opponent in the scoring zones, i.e., face, head, neck, chest, abdomen, back, sides (14). Traditional, sport and Olympic karate competition strands described above differ in their assumptions for interpreting the fight, which is crucial in safety education. Traditional karate teaches decision-making to resolve a fight through a single technique. Sport karate teaches a high degree of manoeuvrability. And Olympic karate develops dynamic techniques of varying levels of difficulty. Therefore, when training young people, they should be given opportunities to confront in all three karate strands so that they learn to adapt their actions to the situation.

It is worth noting that in style and sport karate the fights can be held in the following systems: semi contact, knockdown, full contact, mix fighting; and in Olympic karate they are held only in semi contact. The semi contact system is characterised by limited contact (especially on the head), high technical correctness, speed and intermittent point fighting. The aim is to gain a point advantage over the opponent (15). The knockdown system, on the other hand, emphasises competition with techniques executed with full force. What counts is the effect the technique has on the opponent, but attacking the opponent's head with the upper limbs is prohibited. The aim is to knock down the opponent, which is interpreted as their 3-second inability to continue fighting (16). The full contact system, on the other hand, is characterised by competition using techniques performed with full force on the opponent. It is a combination of the knockdown and semi-contact systems. Therefore, the aim is to win by knocking down the opponent or, in the absence of this, the victory over the opponent is determined by the number of points scored (17). And there is a similar philosophy of confrontation in the mix fighting system, except that apart from the use of techniques performed with full force on the opponent, this system allows for bringing down to the ground and ground fighting. A fight can be won by knocking down the opponent or by correctly applying levers to the opponent and forcing him to submit, and in other cases the victory over the opponent is determined by the number of points scored (18).

These different rules of competition are widely applicable in safety education. But their effect depends on a comprehensive approach. Therefore, young people should train karate by going through all systems. This should depend on the seniority and age of the trainees. Thus, in the initial stage of combat proficiency training, the semi-contact system is justified because it is the safest and develops physical fitness. Then the knockown system verifies the skills of fighting in close contact, delivering and receiving blows and emphasises stamina and strength. The full contact system creates opportunities to simulate an out-of-sport confrontation with full use of motor potential, while the mix fighting system additionally verifies the ability to force an opponent to submit. With the above in mind, young people should gradually be given opportunities to verify offensive and defensive actions in all systems, so that they learn different fighting strategies and anticipation.

## Benefits of karate as a martial art

Approaching karate through a martial art perspective refers to the physical, mental and spiritual development of practitioners through the psycho-physical experience of health-oriented martial training. Here, the martial approach is understood as an undesirable, anxious and contradictory state of mind (19). This adds a highly moral dimension to the practice of karate. Therefore, the perception of traditional values and the internalisation of karate principles is manifested in a distinctive lifestyle of its practitioners referred to as karate culture (9). They are associated with a dojo (karate practice place) represented by a specific master. The master-disciple relationship is built on pedagogical, psychological and sociological principles (20). A systemic approach is emphasised, with a link between individuals, social roles and structures functioning in the group. The status hierarchy depends on cultural and ethical/moral criteria, such as possession of a degree, skills, knowledge, seniority and personality (21). In the dojo, the emotional approach to training, discipline and respect for authority are important. In this sense, karate refers to the control of the younger by the older ranks and their mutual responsibility for safety and order, but also the consequences of maintaining mental hygiene, knowledge of first aid, relaxation exercises and excelling in action strategies (22).

Karate as martial art emphasises the principle of non-violence. It carries a message of peace, avoiding confrontation, stopping conflict, or stopping fighting (23). A trained karateka is more likely than the average person to carry out self-defence, so he or she does not need to prove it if it is possible to avoid a physical confrontation. Furthermore, engaging in a physical confrontation indicates that reason and intelligence have lost−all rational means of solving the problem have failed−so it is appropriate to develop and improve one's life through karate. The aim here is to train martial techniques and adhere to ethical norms for psycho-physical improvement and self-realisation (24). As a result, karate as martial art has beneficial educational influences in safety education. Particularly valuable are the direct forms of transfer of knowledge and skills from master to student, respect for degrees and authorities, respect for moral norms, but also the formation of self-control, self-discipline and the emotional zone of the practitioners. Technical excellence and high physical fitness of the practitioners are also noted, which is the common denominator of karate culture, physical culture and education for safety.

## Benefits of karate as a self-defense system

It is important to note that karate, as a self-defence system, out of the whole stock of combat techniques uses only those that are practical and useful in non-sporting life or health-threatening situations. It focuses on simple movements, using natural human reflexes, in a way that is uncomplicated and accessible to everyone. The karate trained as a self defence system is exclusively for practical applications in non-sport combat. Therefore, the main idea here is to deprive the attacker of the ability to continue fighting. Emphasis is placed on a firm stance during conflict (25). What counts is acting effectively with as much security as possible. The target is primarily the vital points of the opponent. All techniques are geared towards exerting maximum effect on the opponent (26).

Strikes, kicks, pushes, uppercuts, throws and levers are used in combat. In addition, the karate as a self-defence system shapes the skills of anticipating threatening situations, avoiding them, being conscious of the mind, thinking rationally and acting under stress (27). It refers to real situations and effective actions. At the heart of this are the skills of self-defence and defensive action in the situation of a terrorist attack or in a state of war. Therefore, this perspective is important in the education for security of young generations, but it must follow adequately the psychosocial development of the practicioners, as the last stage of training (28). It must be preceded by the upbringing of practitioners through karate as a combat sport and traditional values of karate as a martial art. Otherwise, it may develop antagonistic attitudes among young people (29).

## Discussion

Karate training contributes to the psycho-physical development of adepts of this combat sport, martial art and self-defence system in one (30). This is an important starting point for shaping the behaviour of young people within security education. Nonetheless, karate is also an excellent means of training also those who, wishing to serve the state and society, link their future with work in the formations of the law and order system, where service tasks involve providing security and opposing threats.

Karate is a unique combat method, as its also contains clear references to utilitarian tasks undertaken in the face of serious threats (31). It is within its framework that adepts master bunkai kata (32). This is a set of interpretations of combat blueprints relating to self-defence and fitness. Each blueprint has two options for defence: against an armed and unarmed attacker (33). In addition, adepts acquire control of their own techniques, which is important for practitioner safety (34). Karate training also allows for the acquisition of near-perfect belaying and falling skills, and is an excellent way for women to increase self-defence proficiency (35).

In general, karate can be considered as a means whose application significantly improves the quality of security education. The values of karate as a combat sport, martial art and self defence system contribute to the formation of pro-social values for the security of the state and its citizens. Karate can develop the ethical, motivational and performance spheres of young people (36). The consequence is the recognition of karate as an enriching element of education for security. Therefore, the role of karate coaches should be perceived much more broadly than before (37). In this sense, the karate coach becomes an educator and teacher functioning within youth safety education. He or she thus contributes to shaping their attitudes and behaviour. The safety youth and their social environment may depend on their own behaviour (38). Therefore, it is right that the awareness of the importance of the role of karate coaches for the safety of the state and its citizens should accompany both the society, the developers of safety education and the persons concerned (39).

This highlights the important role of karate in the interpersonal space. It is now an Olympic sport and the most popular martial art in the world (9). The multiplicity of karate styles has contributed to this, and it should be seen as a strength rather than a weakness of the discipline. Karate is recognised globally and among different generations precisely because of its stylistic pedigree. But on the other hand, only treating karate as a whole translates into an all-round action and is applicable to safety education. Accordingly, the utilitarianism of karate in safety education refers to proficiency: in traditional, sport and Olympic strands; in semi contact, knockdown, full contact, mix fighting systems; in ethics, through self-improvement and self-realisation; and in action, through avoiding and anticipating dangers and behaving in times of danger.

## Conclusions

The use of karate in youth safety education is legitimate because this fighting method is based on the values of combat sport, martial art and self defence system. Through karate applied as a combat sport, young people function in a training regime and acquire proficiency in sport fighting according to specific rules. Through karate applied as martial art, young people self-improve their health, develop their corporeality by repeatedly repeating movement tasks and shape their minds by overcoming their own weaknesses and limitations. Through karate applied as a self-defence system, young people acquire proficiency that prepares them for confrontation in defence of their own or others' lives. Taking these three perspectives, karate is an effective means in educating young people to safety.

## Data Availability

The original contributions presented in the study are included in the article/Supplementary Material, further inquiries can be directed to the corresponding author.
